# Enhanced Recovery After Surgery (ERAS) Outcomes After Liver and Pancreas Surgery Based on Regional Perioperative Pain Modalities

**DOI:** 10.7759/cureus.109129

**Published:** 2026-05-18

**Authors:** Megan L Sulciner, Jiping Wang, Mark Fairweather, George Molina, Thomas E Clancy

**Affiliations:** 1 Department of Surgery, Brigham and Women's Hospital, Harvard Medical School, Boston, USA; 2 Department of Surgical Oncology, Brigham and Women's Hospital, Harvard Medical School, Boston, USA

**Keywords:** eras, liver surgery, on-q pumps, pain score, pancreas surgery

## Abstract

Introduction: ON-Q pumps, or surgically placed wound catheters, provide analgesia without the side effects of epidural catheters. This study aimed to evaluate perioperative outcomes, including total postoperative oral morphine milligram equivalents (MMEs), perioperative intravenous (IV) fluid volume, and maximum patient-reported pain scale values, in pancreatic or hepatic resection patients on an Enhanced Recovery After Surgery (ERAS) pathway with ON-Q pumps versus other perioperative pain modalities.

Methods: Patients undergoing ERAS protocol pancreatic or hepatic resection at our institution from January 2019 to March 2022 were included. Patients were categorized based on ON-Q pump, epidural, or transversus abdominis plane (TAP) block/local anesthetic. Total postoperative oral morphine milligram equivalents (MMEs), perioperative intravenous (IV) fluid volume, and maximum patient-reported pain scale values were analyzed.

Results: Of 537 included patients, 249 (46%) received an epidural, 116 (22%) received an ON-Q pump, and 172 (32%) received a TAP block/local anesthetic. ON-Q pump patients required significantly less IV fluids (2,933 mL) compared to 3,379 mL for epidural and 3,278 mL for TAP block/local anesthetic patients (p=0.0037). There was no significant difference in MME requirement or pain scores across pain modalities.

Conclusions: ON-Q pump patients had significantly lower IV fluid requirements, though similar pain scores and MME requirements as epidurals or TAP blocks/local anesthetics. The use of ON-Q pumps as a main perioperative pain modality should be considered for ERAS pathway patients.

## Introduction

Enhanced Recovery After Surgery (ERAS) pathways are evidence-based protocols designed to expedite postoperative recovery and provide cost-effective care [1]. ERAS pathway guidelines have now been implemented by several subspecialty surgical societies for use after major abdominal surgeries such as colorectal, gynecologic, and bariatric, as well as liver and pancreas surgeries [2-6]. Fundamental aims of the ERAS pathway include the minimization of narcotic pain medication and goal-directed intravenous (IV) fluid use [1]. Liver and pancreas surgeries are often complex, and optimizing remains an area of ongoing investigation [5,6].

The use of epidurals as a perioperative pain modality is common both within ERAS pathways and after liver or pancreatic resections [5,7]. Particularly given the morbidity of opioid-related adverse events and the current opioid epidemic, the use of epidurals provides a durable alternative to narcotic pain medications in the perioperative period [8,9]. Notably, epidurals have demonstrated comparable, if not improved, postoperative pain after liver or pancreas surgery to opioid pain medication [10,11]. However, epidurals can lead to hemodynamic changes, namely, hypotension, and subsequently can be associated with aggressive fluid resuscitation [12]. Given that, within the ERAS pathway, postoperative intravenous fluids are projected to be discontinued within 24 hours, these hemodynamic changes can lead to deviations from the ERAS pathway [1]. Thus, a perioperative pain modality that provided adequate pain control, without requiring increased narcotic use, and maintained euvolemia could be an ideal adjunct therapy within ERAS pathways.

ON-Q pumps are surgically placed wound catheters that deliver a constant anesthetic infusion in the preperitoneal or transversus abdominis plane (TAP). Additionally, ON-Q pumps are not associated with hemodynamic changes. ON-Q pumps have demonstrated equivalent pain control as epidurals after major abdominal surgery [13,14]. However, the effect of ON-Q pumps as a perioperative main modality on ERAS pathway outcomes, specifically patient-reported pain scores and fluid management, remains unknown. The aim of this study was to evaluate outcomes, including total postoperative oral morphine milligram equivalents (MMEs), perioperative intravenous (IV) fluid volume, and maximum patient-reported pain scale values, in patients undergoing pancreatic or hepatic resection on an ERAS pathway with ON-Q pumps at our institution.

## Materials and methods

This was a prospective, single-institution study in which we identified all patients undergoing pancreatic or hepatic resection at our institution from January 2019 to March 2022. Patients undergoing pancreatic or hepatic resection via our institution's Enhanced Recovery After Surgery (ERAS) protocol, including for both benign and malignant disease, were included. Our institutional ERAS pathway for patients undergoing pancreatic or hepatic resection includes preoperative carbohydrate loading with a carbohydrate-rich drink, followed by two hours nil per os prior to surgery, intraoperative goal-directed fluid therapy, opioid-sparing analgesia intraoperatively and postoperatively, and early mobilization postoperatively.

The perioperative period was defined as postoperative day (POD) 0 through POD3. Perioperative variables collected included total intravenous (IV) fluid volume administered, total morphine milligram equivalents (MMEs), and patient-reported pain scores on a 10-point scale. The patient's reported pain score was based on a 10-point scale over the perioperative period. Patients with missing data for the variables of interest were also excluded.

Patients were categorized based on anesthetic pain modalities: epidural, ON-Q pump, and TAP block or local anesthetic. All perioperative pain modalities complied with our ERAS protocol. Epidurals were placed by the anesthesia team preoperatively. ON-Q pumps were placed intraoperatively by the operating surgeon prior to skin closure. Local anesthetic was provided by the operating surgeon prior to skin closure, and TAP blocks were placed by the operating anesthesia team after skin incision closure, though prior to extubation. The decision for which perioperative pain modality to implement was surgeon-dependent.

ON-Q pump placement was incision-dependent. For subcostal incisions, the wound was closed in layers with 5-inch or 7.5-inch catheters placed through separate stab incisions into the posterior rectus sheath/transabdominal plane (TAP) laterally or into the preperitoneal plane for re-operative cases. For midline incisions, ON-Q pumps were placed through separate stab incisions into the preperitoneal plane. ON-Q pumps were filled with 1 mg/kg bupivacaine without epinephrine with a maximum volume of 60 mL.

Total MME and perioperative IV fluid volume were calculated as median with 95% confidence interval (CI). Patient-reported pain scores were calculated as the median during the perioperative period (POD0-POD3). Statistical analysis was conducted via the Kruskal-Wallis test. All analyses were performed using GraphPad Prism statistical software version 9.4.0 (GraphPad Software LLC, Boston, MA). Given that this was a quality improvement study, no prior approval by the institutional review board was required per policy by our institution.

## Results

A total of 537 patients who underwent liver or pancreas surgery at our institution were identified for inclusion in this study. All patients were placed on our institution's ERAS pathway. Patients who underwent pancreatic resection comprised 59% (n=318), and patients who underwent liver resection comprised 41% (n=219) of the cohort (Table 1). Patients were then categorized based on perioperative pain modality, including ON-Q pumps, epidural, or TAP block/local anesthetic. A total of 249 patients (46%) received an epidural, 116 patients (22%) received surgically placed ON-Q pumps, and 172 patients (32%) received TAP blocks/local anesthetics.

**Table 1:** Patient characteristics and perioperative pain modality distribution. ERAS, Enhanced Recovery After Surgery; TAP, transversus abdominis plane

Characteristic (n=537)	N (%)
ERAS surgery type	
Pancreas	318 (59)
Liver	219 (41)
Perioperative pain modality	
Epidural	249 (46)
ON-Q pump	116 (22)
TAP block/local anesthetic	172 (32)

**Figure 1:**
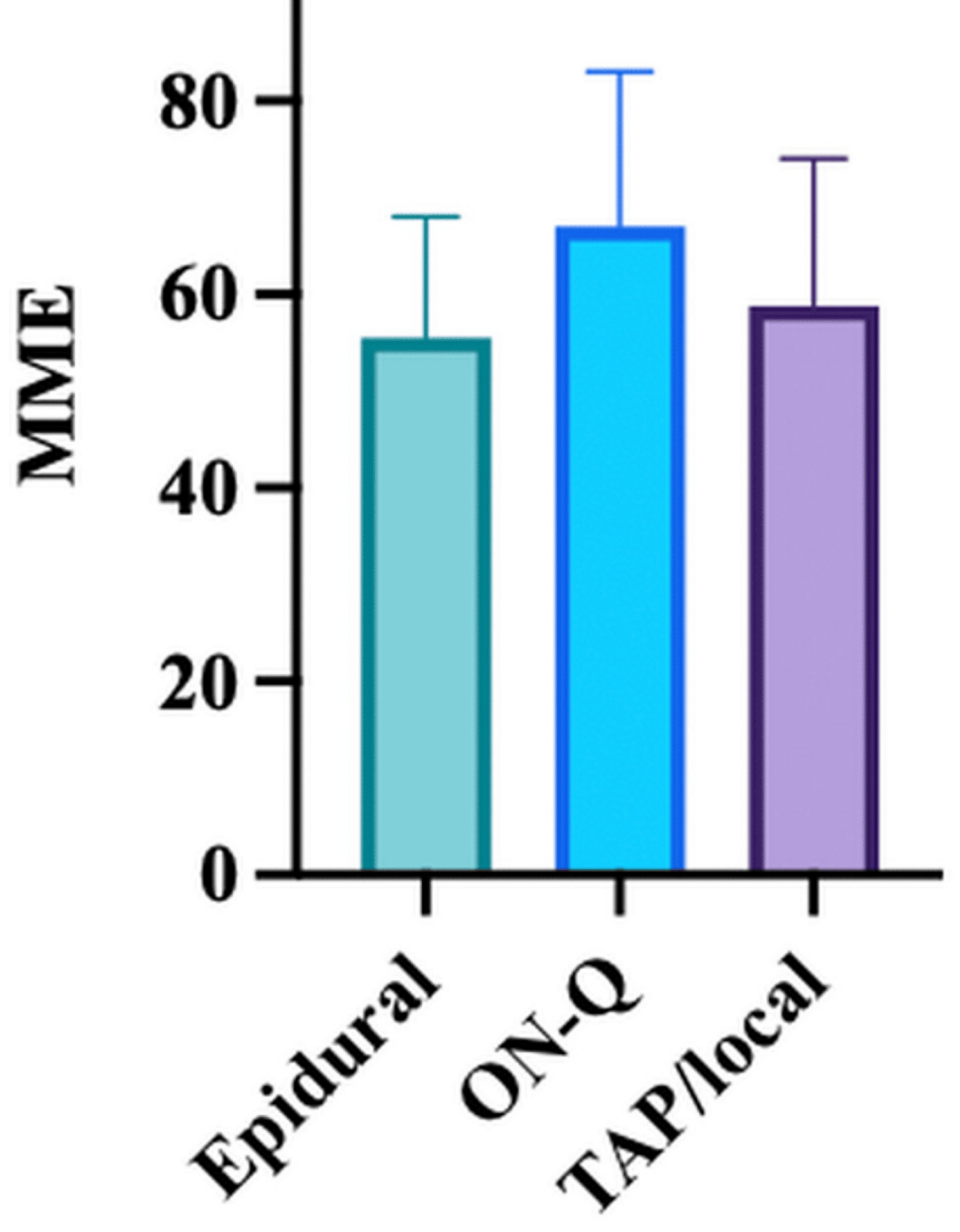
Perioperative morphine milligram equivalent (MME) requirement. The median perioperative morphine milligram equivalent (MME) requirement was similar across the perioperative pain modalities, with 56 MME (95% CI: 39.5-68.0) requirement for epidural, 67 MME (95% CI: 51.0-83.0) for ON-Q pumps, and 59 MME (95% CI: 39.0-74.0) for transversus abdominis plane (TAP)/local (p=0.13). CI: confidence interval

From postoperative day 0 (POD0) to postoperative day 3 (POD3), total perioperative morphine milligram equivalents (MMEs) required per patient in each pain modality were recorded. Patients who received an epidural required a median MME of 56 (Figure 1). Patients who received ON-Q pumps required a median MME of 67, and patients who received TAP blocks/local anesthetics received a median MME of 59. There was no statistically significant difference between MME requirements based on postoperative pain modality.

**Figure 2:**
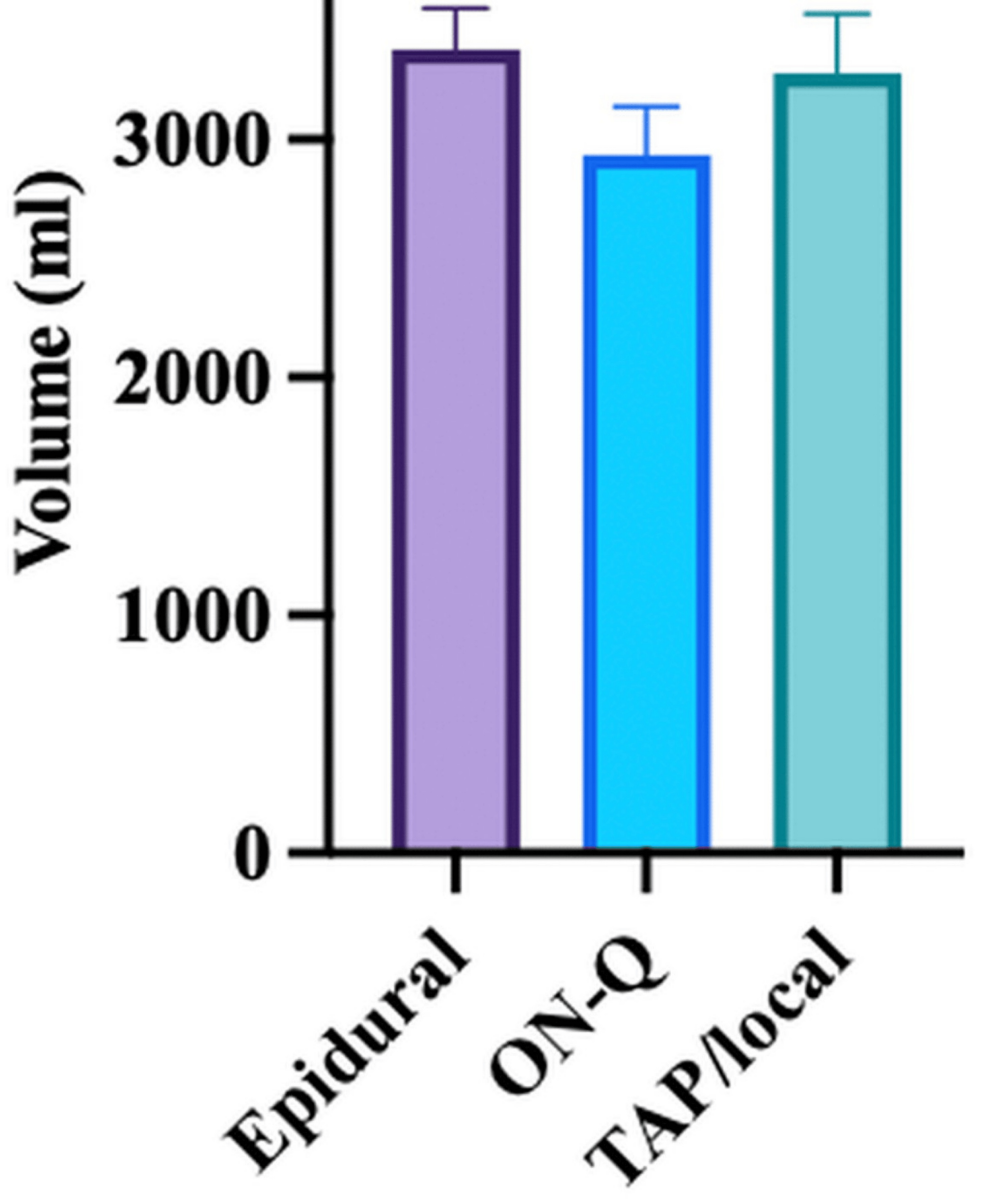
Total perioperative intravenous (IV) fluid volume. The median perioperative intravenous (IV) fluid volume was significantly lower in patients who received ON-Q pumps (2,933 mL; 95% CI: 2,770-3,139) compared to patients who received an epidural (3,379 mL; 95% CI: 3,225-3,552) or transversus abdominis plane (TAP)/local (3,278 mL; 95% CI: 3,134-3,527) (p=0.0037). CI: confidence interval

Total perioperative fluid volume (mL) was also recorded and analyzed based on perioperative pain modality. Patients who received an epidural required a median fluid volume of 3,379 mL, and patients who received TAP blocks/local anesthetics required a relatively similar perioperative IV fluid volume of 3,278 mL (Figure 2). Comparatively, patients who received ON-Q pumps had a median total perioperative fluid volume requirement of 2,933 mL. Patients with ON-Q pumps were found to have a statistically significant lower median perioperative fluid requirement compared to patients who received an epidural (p=0.0037).

**Figure 3:**
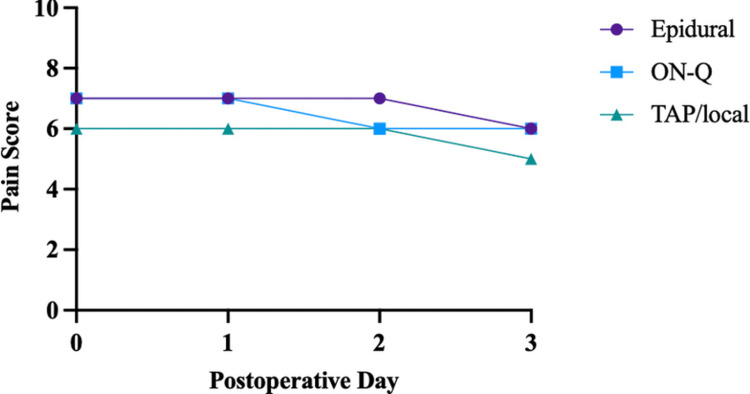
Pain scores from postoperative days (POD) 0-3. Median patient-reported pain score on a scale of 0-10 was similar across the perioperative pain modalities during postoperative day (POD) 0 (p=0.11), POD1 (p=0.18), POD2 (p=0.06), and POD3 (p=0.13). TAP: transversus abdominis plane

Patient-reported pain scores, on a scale of 1-10, were collected from POD0 to POD3 and categorized based on pain modality. On POD0 and POD1, patients who received an epidural or ON-Q pumps reported a median pain score of 7, whereas patients who received TAP blocks/local anesthetics reported a median pain score of 6, though this difference was not statistically significant (Figure 3). On POD3, patients with epidurals continued to have a median pain score of 7, whereas patients with ON-Q pumps and TAP blocks/local anesthetics had median pain scores of 6. On POD3, patients with TAP blocks/local anesthetics reported a median pain score of 5, whereas patients with ON-Q pumps or epidurals reported a median pain score of 6. There was no statistically significant difference between pain scores based on pain modality for POD2 and POD3.

## Discussion

While ERAS pathways have demonstrated benefit in facilitating favorable postoperative outcomes after pancreas and liver surgery, epidural catheter-related hypotension can require significant intravenous fluid support, challenging the goal of judicious fluid administration. We have evaluated outcomes associated with the use of ON-Q pumps, surgically placed wound catheters, as a perioperative pain modality within an ERAS protocol for patients undergoing hepatic or pancreatic resection at our institution. Patients who received ON-Q pumps had similar pain scores and MME requirements in the perioperative period compared to patients who received either an epidural or TAP blocks/local anesthetics. Notably, patients with ON-Q pumps required significantly less intravenous fluids in the perioperative period when compared to the other perioperative pain modalities. This data provides support for the use of ON-Q pumps, in place of epidurals or TAP blocks/local anesthetics, within an ERAS pathway for patients undergoing liver or pancreas surgery.

Poorly managed postoperative pain leads to increased length of stay and readmissions, which ultimately result in overall increased hospital costs [15,16]. Epidurals are a pain modality often integrated into ERAS protocols as a means to provide regional anesthesia while limiting narcotic pain medications [17]. However, the side effects of epidurals, namely, hypotension, may lead to deviation from an ERAS pathway as persistent hypotension after abdominal surgery is associated with increased postoperative complications and extended hospital stay [18]. In comparison, wound catheters have demonstrated quicker functional recovery, as defined by postoperative ambulation, compared to epidurals [19,20]. This observation may be in part due to the fact that patients with wound catheters, such as ON-Q pumps, are less likely to experience hypotension as compared to patients who receive an epidural [19,21].

ON-Q pumps, or similar wound catheters, have demonstrated equivalent pain control as epidurals after several surgeries in various anatomic locations, including the retroperitoneum, chest wall, pelvis, and abdomen [19,22-26]. Wound catheters also have demonstrated similar pain coverage in a pediatric surgery population [27]. In addition, in a randomized controlled trial by Mungroop et al., patients undergoing hepatopancreaticobiliary surgery who had continuous wound infiltration catheters had similar patient-reported pain outcomes when compared to epidurals [14]. In our cohort, we found no significant difference in patient-reported pain scores among patients with ON-Q pumps compared to epidurals in the perioperative period.

Achieving euvolemia after surgery is often challenging, particularly given the biologic changes related to surgical intervention, such as intravascular volume shifts and insensible losses, as well as the hemodynamic changes secondary to anesthetic agents. Avoiding hypovolemia and hypervolemia by goal-directed therapy for intravenous fluid is a main component of ERAS pathways [1,5,6]. The ideal intravenous fluid therapy target for patients undergoing liver surgery remains an area of debate [28,29]. However, for patients who undergo pancreas surgery, minimizing perioperative fluids has been associated with improved postoperative outcomes [30,31]. Our data found that patients who received ON-Q pumps after hepatic or pancreatic resection required a lower median perioperative intravenous fluid (2,933 mL), compared to patients who had an epidural (3,379 mL) or TAP blocks/local anesthetics (3,278 mL). Given that hypovolemia can lead to poor organ perfusion and hypervolemia has been associated with postoperative complications, undoubtedly, the vigilant management of perioperative fluids is required [32-34]. Therefore, one could hypothesize that ON-Q pumps may provide less intrinsic need for additional intravenous fluids that can be associated with epidural-related hypotension.

There are several limitations to this study. First, there was no randomization of perioperative pain modality, as this was a prospective study in which the perioperative pain modality was surgeon-dependent. However, the distribution and representation of pain modalities were relatively similar between patients who received ON-Q pumps or TAP blocks/local anesthetics, with less than half of the patients receiving an epidural. Second, this was a single-institution study, thus potentially limiting applicability to other medical centers. Yet, ERAS pathways are becoming increasingly implemented across medical centers in the United States; moreover, our patient cohort was over a several-year period and included a rather robust number of patients. Additionally, patient pain scores are inherently subjective, though an important aspect within ERAS protocols and an indicator of postoperative pain control. Also, finally, due to this data being collected as part of a quality improvement initiative, the systematic identification and analysis of potential confounding variables, such as patient comorbidities and intraoperative factors, were not performed, representing an important limitation of the study. Future studies should include a randomized controlled trial to further delineate the perioperative outcomes associated with ON-Q pumps compared to other regional perioperative pain modalities.

## Conclusions

Our results demonstrate no significant difference in perioperative MME requirement nor patient-reported pain based on the type of non-narcotic pain medication. Patients with ON-Q pumps had significantly lower IV fluid requirements. The use of ON-Q pumps as a main perioperative non-narcotic pain modality should be considered for ERAS pathway patients given equivalent pain scores and decreased perioperative fluid requirements, in addition to potential physiologic and anesthetic duration benefits compared to epidurals and TAP blocks/local anesthetics alone.
